# Practices and predictors of menstrual hygiene management material use among adolescent and young women in rural Pakistan: A cross-sectional assessment

**DOI:** 10.7189/jogh.12.04059

**Published:** 2022-08-10

**Authors:** Yaqub Wasan, Jo-Anna B. Baxter, Arjumand Rizvi, Fariha Shaheen, Qamaruddin Junejo, Mansoor A Abro, Amjad Hussain, Imran Ahmed, Sajid B Soofi, Zulfiqar A Bhutta

**Affiliations:** 1 Centre of Excellence in Women and Child Health, Aga Khan University, Stadium Road, Karachi, Pakistan; 2 Centre for Global Child Health, The Hospital for Sick Children, Toronto, Ontario, Canada; 3 Department of Nutritional Sciences, University of Toronto, Toronto, Ontario, Canada

## Abstract

**Background:**

In low- and middle-income countries (LMICs), women often use inappropriate materials to manage menstruation, which can threaten their health. Improper practices can also have critical downstream consequences beyond physiologic health, including restricting adolescent girls’ access to academic pursuits.

**Methods:**

We used cross-sectional data collected through a structured questionnaire from the menstruating adolescents and young women 15-23 years of age living in rural Pakistan (n = 25 305). We aimed to describe menstrual hygiene management (MHM) practices and generate a predictive model of the socioeconomic and demographic factors related to the use of MHM materials. Beliefs and barriers around MHM were also summarized. The outcome variable included: those who practiced appropriate and inappropriate MHM practices. Logistic regression was used to generate the predictive model, with results presented as odds ratios (OR) and 95% confidence interval (CI).

**Results:**

Inappropriate MHM practices were reported by 75% (n = 19 006) of participants. The majority 61.9% (n = 15 667) reported using old cloths, 12.6% (n = 3191) used nothing, and 0.5% (n = 136) used old cloth with a sanitary pad. One-fourth of participants reported appropriate MHM material use, including 16.2% (n = 4087) sanitary pads, 8.6% (n = 2167) new cloth, and a few reported using sanitary pads with new cloth 0.2% (n = 45). Inappropriate MHM practices were more common in lowest wealth quintile (OR = 4.41; 95% CI = 2.77-7.01, *P* < 0.0001), followed by those with no education (OR = 3.9; 95% CI = 3.36-4.52, *P* < 0.0001).

**Conclusions:**

The study indicates the need for multi-sectoral efforts to introduce MHM-specific and MHM-sensitive interventions to improve MHM practices, ranging from the availability of low-cost MHM materials to the inclusion of MHM education in school curriculums and within the community platforms.

Menarche is a critical event in a woman’s life, representing a social and physical transition from childhood [1]. If quantified cumulatively, a woman will spend around 6-7 years of her life menstruating [2]. Managing one’s menstrual period appropriately is therefore of great importance. The World Health Organization (WHO) and United Nations International Children's Emergency Fund (UNICEF) define appropriate MHM as the use of clean material to absorb or collect menstrual blood. This also includes changing and disposing of the material at will, in private, and without discrimination. Furthermore, one must have reliable access to appropriate facilities to keep themselves desirably clean [3]. However, LMICs globally can lack the necessary materials and facilities for appropriate menstruation management [4].

UNICEF (2019) has emphasized that multiple Sustainable Development Goals (SDGs) related to health, education, gender equality, and water sanitation and hygiene (WASH) cannot be fully realized without paying due attention to and investing in menstrual health and hygiene [3]. There are several possible consequences to inappropriate MHM. Physiologically, it can increase one’s susceptibility to urinary tract infections [5-7]. Some studies also report an association between secondary infertility and unhygienic MHM practices [8]. There are additionally non-physiological consequences. If a girl has not been adequately informed about menstruation, experiencing menarche for the first time can be traumatic and cause a feeling of distress [9]. Whether a woman is menstruating can also influence her participation in social and religious practices due to cultural norms [2,6,10]. For adolescent girls, school absenteeism during menstruation is broadly reported in various LMIC settings, including Pakistan [9-14]. Such schools typically lack gender-sensitive sanitation facilities to manage menstruation, which can affect adolescent girls’ safety, dignity, and privacy [4,9,13-15]. On a personal level, this can affect girls’ sense of self-esteem and agency [2,6]. From an economic perspective, there can be reduced per capita earning potential, as monthly absenteeism can lead to poor performance and negatively impacts education success [3].

Multiple factors contribute to the way menstrual hygiene is managed. Within LMICs, menstrual hygiene products are frequently reported as not readily available or accessible [11,16]. MHM practices are influenced by cultural beliefs, family environment, and education [17]. Leading causes of inappropriate MHM practices in LMICs include poverty, lack of appropriate sanitation facilities, cost, and access to MHM products [4,10,18].

The use of old clothes to manage menstruation is widely reported across African and South Asian countries [2,10,13,18]. The re-use of the same old cloth over several months has been reported in South Asia [2]. This poses an increased risk of infections and other illnesses [2,6,7]. A systematic review and meta-analysis of 138 studies of menstruation practices in India found that the use of commercial sanitary pads was uncommon among women living in rural locations (Pooled Prevalence (PP) = 32%, 95% CI = 25%-38%, I^2^ = 98.6%, n = 56, *P* < 0.0001) compared to urban (PP = 67%, 95% CI = 57%-76%, I^2^ = 99.3%, n = 38) [10]. Of the few studies that have been conducted in Pakistan, they have found minimal use of appropriate MHM practices during menstruation [9,18].

Among the limited number of published and unpublished research studies assessing MHM, most focus on knowledge and practices. Addressing MHM-related issues has been largely ignored by health managers and policymakers [18]. Collectively, this has led to a call for global action to address the MHM in schools, gaps in understanding, and the development of evidence-based advocacy [4].

Given the limited data and understanding around MHM practices and beliefs in Pakistan, we aimed to understand this among adolescents and young women enrolled in a community-based trial. This paper aims to describe MHM practices and barriers to the use of sanitary napkins and generate a predictive model of factors related to MHM materials. Ideally, findings around MHM practices and determining factors could be utilized by research entities and policymakers could help policymakers design evidence-based interventions to improve the MHM practices among adolescent and young reproductive-age women living in similar settings.

## METHODS

Data were collected from June 2017 to July 2018 as a part of a baseline assessment of a community-based research trial conducted in a rural district, Matiari. This study collaborated with Aga Khan University, Pakistan, and The Hospital for Sick Children, Canada.

Per district health department data, as of June 2020, district Matiari constituted of around 0.8 million population living in about 1800 villages. Nearly half of the population was covered by lady health workers (LHWs), the public sector’s primary outreach health care workforce.

The Matiari empowerment and Preconception Supplementation (MaPPS) Trial primarily aimed to determine the impact of life skills building education (LSBE) and multiple micronutrient supplementation on anemia prevalence and low birth weight (LBW) among adolescent and young women [19,20]. Assessing MHM practices was a secondary outcome of the trial and embedded within the more comprehensive LSBE intervention evaluation framework. LSBE community sessions informed adolescent girls what to expect during menstruation, what happens, and aimed to dissipate stigma. This included discussing appropriate ways to manage menstrual hygiene, consequences of inappropriate cleanliness during menstruation, and general good personal hygiene management practices. The debate was delivered in the community using trained LHWs once a month. However, we have used cross-sectional data on MHM collected upon enrolment in the study (i.e., before exposure to the intervention).

The sample was based on the main MaPPS trial sample size requirement, which was powered to observe a 25% relative reduction in LBW [19]. In total, 25 447 adolescent and young women consented to participate. Of them, 142 participants had reportedly not experienced menarche; thus, they were excluded from this analysis. We did post-hoc power calculations using PASS software under logistic regression procedure [21] on 25 305 menstruating participants, which showed that this study has more than 90% power to detect an odds ratio of 1.5 and above with 95% level of confidence.

Trained female data collectors administrated a structured questionnaire to participants at their homes. This questionnaire included questions consistent with Pakistan demographics and health survey (PDHS) [22], such as demographic data and individual characteristics (e.g., age, marital status, and education), as well as additional questions regarding MHM (age at menarche, MHM practices, source of information on menarche and perceived barriers towards the use of sanitary napkins). Institutional ethics boards approved the study at Aga Khan University and The Hospital for Sick Children, and the National Bioethics Committee in Pakistan. The trial was registered on ClinicalTrials.gov (Identifier: NCT03287882).

### Statistical analysis

We summarized categorical variables as frequency and percentages and non-normally distributed continuous variables as medians and interquartile ranges (IQR). The differences between those who practiced appropriate and inappropriate MHM were compared using non-parametric Wilcoxon rank sum test and χ^2^ test for continuous, and categorical variables respectively.

Univariate analyses between the outcome and predictor variables were first assessed, with results presented as odds ratios (OR), 95% confidence interval (CI), and *P*-values. All variables for which *P* < 0.25 were considered for inclusion in a multivariate analysis from the univariate analyses. A stepwise backward elimination method was applied, and variables that retained significance (*P* < 0.05) were maintained in the final multivariate model. Data were analyzed using STATA version 15.0 (Stata Corporation, College Station, TX, USA).

A bivariate categorical variable for MHM practice was generated based on the method participants reported using to manage their period while menstruating. Women who reported the use of sanitary pads (an absorbent item worn by a woman while menstruating to absorb the blood flow from her vagina that is usually disposable and thrown into the garbage after use) and new cloth (strips of new fabric, often cotton or flannel, used to absorb the blood flow from a woman’s vagina) during menstruation were categorized as practicing “appropriate MHM.” Women who used other materials (old cloth (strips of rags), additional material, and nothing) during menstruation were categorized as practicing “inappropriate MHM.” To generate a predictive model of MHM practices, we considered predictors in the literature and appeared within the MaPPS Trial data set. Associations between demographic, household, and individual factors and MHM practices were evaluated using logistic regression. These included living location (rural (village)/urban (township)), socioeconomic status (SES), participant age, education level, occupation, marital status, religion, decision maker about what to use during menstruation, and hand-washing index. Using standard household indicators based on SES, wealth quintiles (poorest, poorer, middle, more prosperous, and most prosperous) were generated [23]. A cumulative score was calculated for handwashing from the number of times a participant washed her hands at set times, including before preparing food, before eating, and after toilet use. Scored ranged from 0-3: “0” meant no handwashing was observed in the three situations, whereas “3” reflected handwashing in all scenarios.”

## RESULTS

### Menstrual hygiene management material use

Overall, 25% (n = 6299) of participants reported the use of an appropriate MHM material, although materials use was variable, including sanitary pads 16.2% (n = 4087), new clothes 8.6% (n = 2167), and sanitary pad with new fabric 0.2% (n = 45). The majority of participants reported inappropriate use of MHM material; essentially, 61.9% (n = 15 667) of participants said they used old cloth, some 12.6% (n = 1391) reported using no material to manage their most recent menstrual period, and a few reported using old 0.5% (n = 136) cloth with sanitary pads. There was no reported tampon use (**Table 1**).

**Table 1:** Material used to manage blood flow while menstruating (n, %)

	Appropriate MHM (n = 6299)	Inappropriate MHM (n = 19006)	Total (n = 25305)	*P*-value
Sanitary pad	4087 (64.88)	—	4087 (16.15)	<0.0001
New cloth	2167 (34.40)	—	2167 (8.56)	
Old cloth	—	15 667 (82.43)	15 667 (61.91)	
Sanitary pad + new cloth	45 (0.71)	—	45 (0.18)	
Sanitary pad + old cloth	—	136 (0.72)	136 (0.54)	
Used nothing	—	3191 (16.79)	3191 (12.61)	
Other	—	12 (0.06)	12(0.05)	

MHM – management of menstrual hygiene

Except for age, there were differences in demographic characteristics between those who practiced appropriate or inappropriate MHM (**Table 2**). However, there was no difference in MHM material use between adolescents and young women (*P* = 0.369).

**Table 2:** Demographic and individual's characteristics of study participants, disaggregated by MHM practice

Continuous data are presented as median (IQR) and categorical data given as n (%)				
n	**Adequate MHM** (n = 6299)	**Inadequate MHM** (n = 19 006)	**Total** (n = 25 305)	***P*-value**
**Demographic information:**				
Location:				
Rural	2340 (37.15)	10 942 (57.57)	13 282 (52.49)	<0.0001
Urban	3504 (55.63)	6384 (33.59)	9888 (39.08)	
Semi-urban	455 (7.22)	1680 (8.84)	2135 (8.44)	
Socio economic status (SES):				
Poorest	391 (6.21)	4120 (21.68)	4511 (17.83)	<0.0001
Poor	613 (9.73)	4200 (22.10)	4813 (19.02)	
Middle	879 (13.95)	4229 (22.25)	5108 (20.19)	
Rich	1559 (24.75)	3781 (19.89)	5340 (21.10)	
Richest	2857 (45.36)	2676 (14.08)	5533 (21.87)	
Individual information:				
Age median (IQR)	18.63 (16.78-20.69)	18.65 (16.76-20.74)	18.63 (16.76-20.72)	0.738
Age categories:				
15-19 y	3613 (57.36)	11 024 (58.0)	14 637 (57.84)	0.369
19-23 y	2686 (42.64)	7982 (42.0)	10 668 (42.16)	
Age at marriage, median (IQR), n	17 (16,18) n = 1264	17 (15,18) n = 4567	17 (15,18) n = 5831	<0.0001
Education:				
None	1383 (21.96)	9922 (52.20)	11 305 (44.67)	<0.0001
Primary	1289 (20.46)	4737 (24.92)	6026 (23.81)	
Secondary	1883 (29.89)	3225 (16.97)	5108 (20.19)	
Higher secondary/university	1744 (27.69)	1122 (5.90)	2866 (11.33)	
Occupation:				
Within the home	3118 (49.50)	8912 (46.89)	12 030 (47.54)	<0.0001
Unskilled manual labor	415 (6.59)	4010 (21.10)	4425 (17.49)	
Skilled manual labor	875 (13.89)	4240 (22.31)	5115 (20.21)	
Others (professional & unemployed)	149 (2.37)	97 (0.51)	246 (0.97)	
Student	1742 (27.66)	1747 (9.19)	3489 (13.79)	
Marital status:				
Never married	5033 (79.90)	14 432 (75.93)	19 465 (76.92)	<0.0001
Married	1224 (19.43)	4458 (23.46)	5682 (22.45)	
No longer married	42 (0.67)	116 (0.61)	158 (0.62)	
Religion:				
Muslim	5927 (94.09)	16 996 (89.42)	22 923 (90.59)	<0.0001
Other	372 (5.91)	2010 (10.58)	2382 (9.41)	
Decision-maker about what to use during menstruation:				
Participant	5118 (81.25)	16 418 (86.38)	21 536 (85.11)	<0.0001
Mother	565 (8.97)	843 (4.44)	1408 (5.56)	
Participants + others	597 (9.48)	1678 (8.83)	2275 (8.99)	
Others	18 (0.29)	47 (0.25)	65 (0.26)	
No response	1 (0.02)	20 (0.11)	21 (0.08)	
Hand washing index:*				
0	15 (0.24)	282 (1.48)	297 (1.17)	<0.0001
1	120 (1.91)	756 (3.98)	876 (3.46)	
2	779 (12.37)	2511 (13.21)	3290 (13)	
3	5385 (85.49)	15457 (81.33)	20842 (82.36)	

MHM – management of menstrual hygiene, IQR – interquartile range, y – year

*Cumulative score of total frequencies for handwashing (ie, before preparing food, before eating, and after using the toilet).

### Participants’ characteristics

Participants reported median (IQR) age was 18.6 (16.8-20.7) years, and on average, they experienced menarche at 13 (12,14) years. Twenty-three percent of participants were married, age at marriage was 17 (15,18) years. Around half of the study participants 52.5% (n = 13 282) were from rural villages. Most participants were Muslim 90.6% (n = 22 923), and 44.7% (n = 11 305) reported no formal education (**Table 2**).

### Univariate and multivariate analysis

As there was no difference in the MHM practices between adolescent and young women (*P* = 0.369), all participants’ data were considered together. Univariate analysis showed that the likelihood of inappropriate MHM practices was significantly higher if one belonged to a lower wealth quintile (OR = 11.24; 95% CI = 7.17-17.79, *P* < 0.0001), followed by having no education (OR = 11.15; 95% CI *=* 8.80-14.12, *P* < 0.0001). Additional factors significantly associated with MHM practices included area of domicile, occupation one belonged to, marital status, religion, decision-making authority about what to use for MHM, and handwashing score (**Table 3**).

**Table 3:** Factors associated with inadequate menstrual hygiene management practices

	Univariate model			Multivariate model		
	**OR**	**95% CI**	***P*-value**	**OR**	**95% CI**	***P*-value**
**Demographic information**						
Location:						
Rural	2.56	1.69-3.88	<0.0001	—	—	—
Urban	Ref.					
Semi-urban	2.02	0.92-4.44	0.078	—	—	—
SES						
Poorest	11.24	7.17-17.79	<0.0001	4.41	2.77-7.01	<0.0001
Poor	7.31	5.24-10.19	<0.0001	3.52	2.64-4.69	<0.0001
Middle	5.14	3.83-6.88	<0.0001	2.96	3.35-3.74	<0.0001
Rich	2.59	2.04-3.28	<0.0001	1.85	1.53-2.23	<0.0001
Richest	Ref.			Ref.		
**Individual information**						
Education:						
None	11.15	8.80-14.12	<0.0001	3.9	3.36-4.52	<0.0001
Primary	5.71	4.32-7.54	<0.0001	2.95	2.46-3.53	<0.0001
Secondary	2.66	2.26-3.12	<0.0001	1.98	1.76-2.22	<0.0001
Higher secondary/university	Ref.			Ref.		
Occupation:						
Within the home	Ref.			Ref.		
Unskilled manual labor	3.38	2.49-4.57	<0.0001	1.55	1.07-2.23	0.018
Skilled manual labor	1.69	1.30-2.20	<0.0001	1.37	1.12-1.67	0.002
Others (professional & unemployed)	0.23	0.17-0.30	<0.0001	0.58	0.46-0.72	<0.0001
Student	0.35	0.30-0.40	<0.0001	0.74	0.68-0.80	<0.0001
Marital status:						
Never married	0.79	0.68-0.90	0.001	1.18	1.05-1.32	0.005
Married	Ref.			Ref.		
No longer married	0.76	0.50-1.14	0.186	0.69	0.43-1.13	0.142
Religion:						
Muslim	Ref.					
Other	1.88	1.18-2.99	0.007	—	—	—
Decision-maker about what to use during menstruation:						
Participant	2.15	1.03-4.45	0.040	2.56	1.10-5.96	0.029
Mother	Ref.			Ref.		
Participants + others	1.88	0.92-3.82	0.080	2.44	1.07-5.56	0.034
Others/no response	2.36	1.43-3.90	0.001	2.22	1.18-4.18	0.014
Hand washing index:						
0	6.55	3.64-11.77	<0.0001	3.12	1.80-5.42	<0.0001
1	2.19	1.65-2.92	<0.0001	1.89	1.37-2.61	<0.0001
2	1.12	0.89-1.42	0.335	1.37	1.15-1.64	<0.0001
3	Ref.			Ref.		

Ref. – reference category, OR – odds ratio and CI – confidence interval, SES – socio-economic status

Within the multivariate model, religion and living rurally did not remain as a significant predictive factor. The largest effects were observed for SES (OR = 4.41; 95% CI = 2.77-7.01, *P* < 0.0001) and having no education (OR = 3.9; 95% CI = 3.36-4.52, *P* < 0.0001). With progression from lower to higher SES categories, a clear gradient exists across quintiles for the practice of appropriate MHM. Improper hand hygiene was also associated with greater inappropriate MHM (OR = 3.12; 95% CI = 1.80-5.42, *P* < 0.035) (**Table 3**).

### Barriers to appropriate MHM

The cost was reported to be one of the most significant barriers to the use of sanitary pads by participants 48.7% (n = 9275). Lack of availability and awareness about appropriate materials with which to manage MHM were reported by 17.4% (n = 3300) and 12.1% (n = 2294) of participants, respectively. Cultural and religious reasons for not using sanitary pads were reported by 21.9% (n = 4155) of participants, including shyness to ask someone to bring it from the market; participants’ lack of comfort with using sanitary pads; and that throwing out a sanitary napkin immersed with blood was perceived to be a sinful act. Some participants also indicated that the lack of sanitation facilities to dispose-off sanitary pads was a barrier (**Table 4**).

**Table 4:** Reasons (n, %) for not using sanitary pads

Data presented as n (%)					
	**Affordability**	**Knowledge**	**Availability**	**Cultural /Religious reasons**	**Total**
	9257 (48.71)	2294 (12.07)	3300 (17.36)	4155 (21.86)	19 006
**Demographic information**					
Location:					
Rural	5209 (47.61)	1506 (13.76)	2201 (20.12)	2026 (18.52)	10 942
Urban	3211 (50.30)	485 (7.60)	953 (14.93)	1735 (27.18)	6384
Semi-urban	837 (49.82)	303 (18.04)	146 (8.69)	394 (23.45)	1680
SES:					
Poorest	2082 (50.53)	1062 (25.78)	495 (12.01)	481 (11.67)	4120
Poor	2262 (53.86)	592 (14.10)	694 (16.52)	652 (15.52)	4200
Middle	2201 (52.05)	364 (8.61)	855 (20.22)	809 (19.13)	4229
Rich	1826 (48.29)	197 (5.21)	756 (19.99)	1002 (26.50)	3781
Richest	886 (33.11)	79 (2.95)	500 (18.68)	1211 (45.25)	2676
**Individual information**					
Age:					
15-19 y	5306 (48.13)	1386 (12.57)	1907 (17.3)	2425 (22.00)	11 024
19-23 y	3951 (49.50)	908 (11.38)	1393 (17.45)	1730 (21.67)	7982
Education:					
None	5028 (50.68)	1774 (17.88)	1513 (15.25)	1607 (16.20)	9922
Primary	2297 (48.49)	387 (8.17)	874 (18.45)	1179 (24.89)	4737
Secondary	1487 (46.11)	123 (3.81)	682 (21.15)	933 (28.93)	3225
Higher secondary/university	445 (39.66)	10 (0.89)	231 (20.59)	436 (38.86)	1122
Occupation:					
Within the home	4383 (49.18)	888 (9.96)	1547 (17.36)	2094 (23.50)	8912
Unskilled manual labor	1917 (47.81)	955 (23.82)	431 (10.75)	707 (17.63)	4010
Skilled manual labor	2152 (50.75)	401 (9.46)	920 (21.70)	767 (18.09)	4240
Other/professional/unemployed	48 (49.48)	6 (6.19)	16 (16.49)	27 (27.84)	97
Student	757 (43.33)	44 (2.52)	386 (22.10)	560 (32.05)	1747
Marital status:					
Never married	6862 (47.55)	1664 (11.53)	2553 (17.69)	3353 (23.23)	14432
Married	2323 (52.11)	619 (13.89)	731 (16.40)	785 (17.61)	4458
No longer married	72 (62.07)	11 (9.48)	16 (13.79)	17 (14.66)	116
Religion:					
Muslim	8207 (48.29)	1814 (10.67)	3093 (18.20)	3882 (22.84)	16996
Other	1050 (52.24)	480 (23.88)	207 (10.30)	273 (13.58)	2010
Decision-maker about what to use during menstruation:					
Participant	8027 (48.89)	1893 (11.53)	2798 (17.04)	3700 (22.54)	16418
Mother	211 (25.03)	146 (17.32)	310 (36.77)	176 (20.88)	843
Participants + others	1007 (60.01)	226 (13.47)	176 (10.49)	269 (16.03)	1678
Others/No response	12 (17.91)	29 (43.28)	16 (23.88)	10 (14.93)	67
Hand washing index*					
0	110 (39.01)	98 (34.75)	47 (16.67)	27 (9.57)	282
1	375 (49.60)	138 (18.25)	87 (11.51)	156 (20.63)	756
2	1221 (48.63)	309 (12.31)	363 (14.46)	618 (24.61)	2511
3	7551 (48.85)	1749 (11.32)	2803 (18.13)	3354 (21.70)	15457

SES – socio-economic status, y – year

*Cumulative score of total frequencies for handwashing (ie, before preparing food, before eating, and after using the toilet)

### Consequences of menstruation

Slightly less than half of the participants 45.4% (n = 11 501) indicated that their routine activities were restricted due to menstruation. Among the 2147 participants who reported attending school regularly, 22.2% mentioned that they were not going to school while menstruating.

## DISCUSSION

The current literature suggests that various socio-cultural and structural reasons are the basis of why many girls and women living in LMICs often do not practice appropriate MHM [4,10]. Notably, MHM is widely excluded from public infrastructure design and public health promotion campaigns within South Asia. There is limited guidance for health workers [2]. We aimed to understand MHM practices and the predictors and barriers to practicing appropriate MHM among a cohort of adolescent and young women in rural Pakistan.

We found that 25% of participants practiced appropriate MHM, which is lower than figures reported in other regions of Pakistan. However, none were conducted rurally, as was the case in our study [14,18,23]. For example, Michael et al. recently reported 68.7% use of commercially available sanitary pads in a study conducted in Quetta [14]. Mumtaz et al. reported that 50.2% of adolescent girls either use sanitary pads 16.8% or new cloths 33.4% during menstruation in a study conducted in peri-urban Islamabad [24]. In a Karachi-based study, Ali et al. reported that 41.4%, 29.9%, and 21.2% of adolescent girls from private schools, public schools, and out-of-school, respectively, used hygienic MHM materials [18]. Furthermore, findings of a meta-analysis of community-based studies conducted in India showed that a third of adolescent girls used sanitary pads (PP = 32%, 25%-38%, I^2^ = 98.6%, n = 56, *P* < 0.0001) [10]. Collectively, this demonstrates that a more significant proportion of adolescent and young women in Matiari practice inappropriate MHM material to manage menstruation than their peers in other parts of Pakistan and in India.

Affordability is a barrier to the use of appropriate MHM materials across LMICs [10,25-27]. Various studies carried out in parts of Africa and Asia have suggested the suboptimal use of MHM materials is because of the high cost of hygienic absorbents. Most qualitative and quantitative findings are from school-based settings [10,12,18,25-27]. In our community-based study, household wealth had the strongest association with the use of either material during menstruation. Participants who belonged to the poorest wealth quintile were >4 times as likely to practice inappropriate MHM during menstruation than those in the richest quintile. Study results revealed that almost half 48.7% of participants reported cost as a barrier to the use of sanitary napkins. This suggests that producing and accessing affordable hygienic-sanitary material will be critical to improving MHM in the study setting.

Our study findings revealed a robust relationship between having formal education and the appropriate use of menstrual hygiene material, as schooling was significantly associated with higher use of sanitary pads (OR = 3.9; 95% CI = 3.36-4.52, *P* < 0.0001). Because education level increased correspondingly with the wealth quintile, access to appropriate MHM materials may be limited by poverty. A review of studies from Indian settings showed less common use of old cloths to manage menstruation (inappropriate MHM) in the studies carried out in school settings compared to those in the community [10]. Ali et al. also reported greater than 2-fold use of sanitary pads among adolescent girls studying in private school compared to out-of-school participants in Karachi. They emphasized the need to initiate MHM awareness programs beyond school platforms [18]. Together with our current findings, this suggests the importance of community-based platforms to reach out-of-school girls and women, given that the practice of inappropriate MHM among these groups was high.

We found that two-thirds of study participants reported inappropriate MHM. The majority lived in more remote villages within the study catchment area. Consistent with the trends reported in several studies, the univariate analysis suggested that participants who belonged to comparatively urban areas were more likely to practice appropriate MHM OR = 2.56 (CI = 59% = 1.69-3.88, *P* < 0.0001) [5,10]. The impact of living rurally on the use of sanitary products may not be well understood in isolation, as educational attainment and access to the clean products or sanitary pads are higher in urban areas.

Our current findings revealed that about one-fifth of study participants 17.4% lacked access to hygienic MHM material. This is further complicated by study participants’ reported unease in buying sanitary pads from the shops, which male vendors usually run; on top of that, they did not feel comfortable asking their parents to buy MHM materials. The lack of suitable facilities to dispose of sanitary napkins was also identified to restrict the uptake of sanitary pads. Interestingly, a tenth of participants were unfamiliar with sanitary pads; most rural and semi-urban areas belonged to poorer wealth quintiles and lacked formal education. Overall, we believe there is a need to improve knowledge and awareness around the appropriate use of MHM materials, as highlighted by various researchers across settings, to dissipate stigma, spatial restrictions, gender inequalities and enhance school attendance [14,16,26,28].

### Limitations

While we aimed to understand what materials participants within the MaPPS trial used to manage menstruation, we did not investigate the diverse factors that contributed to their decision-making. Study data was limited to self-reported, structured questionnaire information, which does not allow for more nuanced and qualitative data capture that could inform why appropriate menstrual hygiene management practices were low. The inclusion of health managers and market suppliers’ perspectives around MHM materials could have enriched the data and provided more robust recommendations to address the issue. Given the observed effect of menstruation on girls’ school attendance, an assessment of private and appropriate hygiene and disposal facilities in schools could offer greater insight.

### Strengths

This study included data from >25 000 adolescent and young women aged 15-23 years. While most studies on MHM have only focused on adolescent girls enrolled in schools, the current study provides us with an opportunity to expand our understanding of community based MHM practices. Furthermore, the study fills the gap in information on MHM in rural settings within Pakistan. We hope this might serve as a platform for researchers to explore and enable appropriate MHM practices further. The presented evidence may help health managers to design a programmatic set of actions to address the MHM issues of girls and women living in similar settings.

## CONCLUSIONS

Within our assessment, most participants were not found to practice what is considered appropriate MHM. The factors that predicted the use of proper MHM and the barriers reported to inhibit the use of sanitary pads are not unique to this setting and reflect the findings within several other LMICs. There is a need for a synergistic initiative from different sectors to tackle the identified obstacles to MHM adequately. The introduction of MHM-specific and MHM-sensitive interventions ranging from the availability of low-cost MHM materials to the inclusion of MHM awareness in school curriculums and educational materials for use in community platforms can potentially improve MHM. Given the existing culture of silence around menstruation, the educational material should also aim to sensitize the male segments of communities. Moreover, local low-cost production of MHM materials, possibly accompanied by the engagement of local girls and women, could serve to address the MHM-specific barriers and contribute to overall women’s economic empowerment.
